# Telemedicine and Pediatric Care in Rural and Remote Areas of Middle-and-Low-Income Countries: Narrative Review

**DOI:** 10.1007/s44197-024-00214-8

**Published:** 2024-03-13

**Authors:** Yossef Alnasser, Alvaro Proaño, Christine Loock, John Chuo, Robert H. Gilman

**Affiliations:** 1 Milken Institute of Public Health, George Washington University, Washington, DC USA; 2 Pediatric Department, King Saud University, Riyadh, Saudi Arabia; 3 Pediatric Department, BronxCare Health System, Bronx, NY USA; 4 Division of Neonatology, Department of Pediatrics, Children’s Hospital of Philadelphia, Philadelphia, PA USA; 5 British Columbia Children’s Hospital, University of British Columbia, Vancouver, BC Canada; 6 International Health, Johns Hopkins Bloomberg School of Public Health, Baltimore, MD USA

**Keywords:** Telemedicine, Pediatric, LMIC, Rural Areas, Under-5 Mortality and Neonatology

## Abstract

**Objectives:**

Caring for children in low- and middle-income countries (LMIC) can be challenging. This review article aims to explore role of telemedicine in supporting pediatric care in LMIC.

**Methodology:**

A narrative review of existing English and Spanish literature was conducted to assess role of telemedicine to support pediatric care in LMIC.

**Results:**

Beside medical education and direct pediatric care, telemedicine can provide sub-specialties consultations without extra burden on families. Additionally, telemedicine can help in lowering under-5 mortality by supporting neonatal care, infectious illnesses, and non-communicable diseases (NCDs). Telemedicine can be a gate for universal coverage for all children at a lower cost. For over a decade, it has been implemented successfully and sustained in a few LMIC. However, challenges in implementing telemedicine are enormous. Still, opportunities arise by using simpler technology, low-width band internet, smartphones, instant messaging applications and solar energy. COVID-19 pandemic facilitated acceptance and applicability of telemedicine worldwide including LMIC. Nevertheless, governments must regulate telemedicine by issuing policies and ensuring employment of local experts when possible to meet local resources and cultural competency.

**Conclusion:**

Telemedicine has proven successful in improving pediatrics care. Many LMIC should take advantage of this innovation to promote equity and access to high quality pediatric care.

## Introduction

Delivering child and youth healthcare to rural communities can be challenging, especially as it involves specialized care [1, 2]. Members of these communities often need to relocate in order to access pediatric specialized care. Such movements are burdensome financially, physically psychosocially, and emotionally [3]. While this is a global health problem affecting all income countries, developing and poor countries with scarce resources are most impacted. Innovative and creative new care models of pediatric healthcare using telemedicine may overcome access barriers in remote and rural areas [4, 5].

In many low-and middle-income countries (LMICs) the healthcare model promotes primary care in rural areas. Many LMICs depend on new medical graduates or non-physician-clinicians (NPCs) in rural and hard-to-reach areas [4, 5]. The providers taking care of pediatric patients are not always trained in general pediatrics or its subspecialities, requiring the families to relocate for healthcare access. With high poverty rates, many families must make the difficult choice between high financial burden of relocating or remaining closer to home, hoping that their child gets better on their own [6].

Information and communication technologies (ICT) are reshaping healthcare in many ways [7]. Telemedicine is one form of ICT that has an evolving definition and manifesting in various forms [8]. Since it was first described, in 1970, it has advanced with new technologies that have brought medicine closer to the community [9]. The World Health Organization (WHO) defined telemedicine as “the delivery of health care services, where distance is a critical factor, by all health care professionals using information and communication technologies for the exchange of valid information for diagnosis, treatment and prevention of disease and injuries, research and evaluation, and for the continuing education of health care providers, all in the interests of advancing the health of individuals and their communities” [10]. Telemedicine has been implemented in developed countries for decades [11]. It has the potential to have a higher impact in developing countries where resources are scarce, and cost of care is a major determinant of health [12] Many LMICs around the globe adopted different forms of telemedicine which was accelerated by the COVID-19 pandemic [13].

### Why is Telemedicine Needed in Rural and Hard-to-Reach Areas of LMIC?

Telemedicine can improve child and youth healthcare access in remote and rural areas [14]. This can decrease the burden of disease on children and their families while improving their quality of life. This is very important in Sub-Saharan Africa as 40% of populations reside in rural communities while most hospitals and specialists are in big cities [15]. Cost and lack of proper medical transport system make telemedicine sometimes the only option for rural children and their families [16]. This is a major difference between telemedicine in high-income countries and LMIC. It runs concurrently to traditional healthcare models in high-income countries while it might be the only alternative in LMIC [17].

### Can Telemedicine Overcome Child Health Disparities?

With successful implementation of telemedicine programs in developing countries, it seems to be an appealing healthcare model to improve heath equity and equality [18, 19]. However, disparity still exists even within telemedicine. Gender disparity persisted among telemedicine beneficiaries as documented by Rahman et al. [20]. Gender disparity is the difference of gaining basic human rights based on gender [21]. Fewer female children accessing healthcare is a true example of gender disparity and a known global public health concern [22]. Unfortunately, telemedicine has not been able to close the gender gap in accessing healthcare so far. Another gap that might influence lower telemedicine access is poor health literacy [23]. Still, some forms of telemedicine can be employed for health promotion to augment better health literacy and higher access to care [24]. Regardless of health literacy level, digital divide can add more disparities into childcare [25]. Lack of access to modern tele-communication devices or having a device without internet coverage are true examples of digital divide in many LMICs. With high penetration of smartphones in many low-income countries, focusing on phone-based intervention (e.g. mobile health [mHealth]) can overcome digital disparity and has been already used by many providers in LMIC [26–28]. With modernization and the advancement of technology, the digital divide will narrow in the future. Phone-based telemedicine and telemedicine in general can reach technology-savvy groups like adolescents. It has been proven to reach more youths and improve their access to care [29]. However, confidentiality and privacy should be highly respected when delivering telemedicine to serve adolescents [30]. Overall, telemedicine can be a vehicle of change to lower health disparities among children and youths in LMIC as was evident in Brazil’s experience [31].

### What are the Challenges in Implementing and Maintaining Pediatric Telemedicine in LMIC?

Challenges in implementing pediatric telemedicine in developing countries and rural areas are enormous [18, 32, 33]. Network, lack of policy, digital divide and other inadequate infrastructures are the biggest challenges [34]. Low bandwidth telemedicine has been proven effective in overcoming poor network connectivity in Cambodia, Uzbekistan, and Kosovo [16]. However, low bandwidth Internet might make it hard to run certain software and applications. To overcome some of these challenges, simplifying the use of technology and investing in already used applications can be a good alternative. Investing in commercially available multidimensional instant messaging (IM) application as telemedicine platform was successful [35]. Concerns of confidentially with IM can be addressed by adding more privacy safeguards [36]. With the ever-increasing prevalence of smartphones in many LMIC, phone-based telemedicine strategies might be a practical and digitally equitable solutions that could also be cheaper than computer-based alternatives [37]. Phone based applications can provide variable platforms for telemedicine including written consultations, audio or video consults. Choosing audio over video-based telemedicine services can be dictated by insurance type, ethnicity, patients’ income, and level of education as documented in United States [38]. Furthermore, connectivity in low-income countries requires basic technical infrastructure including reliable electricity, mobile coverage and/or internet access, and energy sources. Using solar system can be a cheaper and an ecologically friendly alternative [39].

Identifying providers with pediatric and child-youth health specialty training and understanding the challenges in under-resourced settings is another challenge. The recruitment of international specialists can potentially contribute to harm when lacking understanding of culture, colonialism and other historical traumas, in addition to lack of awareness of the variability of locally available resources [40]. In developed countries, reimbursement and billing is a recurring dilemma with respect to telemedicine [11]. Reimbursement of pediatric providers for telemedicine services in low-income countries might be a future challenge. Currently, most low-income countries depend on volunteering specialists [16]. This can make the service less sustainable. However, there are hopeful examples from LMIC where a telemedicine project was sustained for longer than a decade [41].

Another challenge includes how intrusive telemedicine can be to both traditional medicine and workflow [33]. This can add a burden on rural providers where healthcare worker capacity is already overstretched due to overwhelming provider-to-patient ratios. To incorporate telemedicine in busy clinics with shortage of providers and high patient volume, it has to be adaptable. Asynchronous (store and forward) telemedicine provides more flexibility than real­time telemedicine and can be suitable to non-urgent and primary care cases [42]. Still, real-time (synchronous) telemedicine platforms can help in emergency and critical care pediatric cases for urgent decision-making [43].

### How can Telemedicine Support LMIC Pediatric Providers?

Pediatricians are generally the “cornerstones” of child healthcare [44]. Telemedicine is currently playing a big role in improving care. In the United States the top 5 services of pediatric care via telemedicine include neurology, psychiatry, cardiology, neonatology and critical care [45, 46]. On the other side, primary and specialized pediatric care delivery cannot be centered on pediatricians in LMIC. With fewer primary care doctors and pediatric specialists per capita, new modules of care are needed. An alternative has been in place for several years in Sub-Saharan Africa [47]. NPCs have been delivering primary care in rural areas in many sub-Saharan countries [48]. Their potential has been expanded and scaled up to serve their local public [49] Telemedicine can be a tool to train NPCs and improve their quality of care for all children. COVID-19 has expanded incorporation of telemedicine in medical education [13, 34]. With continuity of care and interaction with experts, NPCs can build their pediatric clinical skills and enhance their knowledge [50]. Telemedicine has many applications to support pediatric care in LMIC. It can enhance neonatal care in low-income countries as neonatal causes are the leading etiologies of under-five mortality [51, 52]. Scope of telemedicine in neonatal-perinatal medicine can be expanded [51] to include resuscitation of newborns via programs such as the Neonatal Resuscitation Program (NRP) or Helping Baby Breathe [53, 54]. In the Neonatal Intensive Care Unit (NICU) it has also been used for ‘tele-rounding’, where the neonatologist does not need to be physically present in the unit, but rounds via a remotely controlled robot, this can be used to help staff and manage neonatal units around the world [55]. Another important aspect of managing preterm newborns includes screening for retinopathy of prematurity, and there are reports that show that telemedicine can be used for this screening [56]. In general, telemedicine has been shown to decrease the need for transferring neonates and children to other institutions, and increases quality of local care that ultimately helps keeps families together in their own communities [57]. Additionally, it can also be used to help improving maternal and antenatal care by improving access and quality of care [58–60].

Beyond the neonatal period, telemedicine can enhance pediatric care and improve decision-making [61]. From radiology support to infectious diseases, the potential of telemedicine extended to support many practitioners in LMIC [62, 63]. Many non-communicable diseases (NCDs) are expected to grow with epidemiological shift in many LMIC [64]. Skills and capacity to care for NCDs, like pediatric cardiology and pediatric oncology, have been enhanced by telemedicine in developed and developing countries [65–67]. Furthermore, telemedicine can be used as tool for digital medical education to boost capacity building and task shifting to improve care and access at a lower cost [68, 69]. Besides lowering costs, telemedicine can promote antibiotic stewardship to suppress multidrug resistant organisms: a real threat for global health [70]. Through telemedicine, unnecessary use of antibiotics can be avoided along with tailoring choice of antibiotics when possible. Furthermore, telemedicine can be a tool for equity besides improving pediatric care. Telemedicine has been used to overcome language barriers and provide interpreter support [71]. Ideally, telemedicine can be a step forward toward universal health coverage for all children [72].

### Can Telemedicine Lower the Cost of Pediatric Care in LMIC?

Despite the challenges in implementing telemedicine in LMIC, it can provide cheaper and cost-effective healthcare models [73, 74]. Telemedicine can overcome shortage of specialized pediatricians and allow higher access of children to specialized service without increasing cost [75]. It can lower direct medical cost in comparison to specialized in-person care by 31% by achieving higher net-savings by higher volume of consultations [76]. Furthermore, cost saving provided by telemedicine comes with gains in quality adjusted life years (QALYs) [75]. Indirect cost savings were documented to include travel costs and potential loss of productivity due to direct medical visits in a high-income country [77]. Those savings might be more meaningful in low-income countries with scarce resources and limited transportation options. With lower cost and ease of access, fear of overutilization of telemedicine is a potential concern to drive healthcare cost higher and overwhelm the healthcare system [78]. However, overutilization by underserved populations should not be a primary concern at this stage and can be easily regulated in the future if proven to be true.

### How has the COVID-19 Pandemic Impacted Pediatric Telemedicine?

COVID-19 pandemic has played a role in expanding telemedicine globally [13]. However, the added impact of COVID − 19 pandemic and its aftermath have disproportionally affected equity-deserving populations globally [79]. Telemedicine can be used as a tool for change, to build back better. Furthermore, COVID-19 pandemic has provided an opportunity to witness potentials of telemedicine [13]. In developed and developing countries, COVID-19 pandemic increased telemedicine acceptability and removed many barriers [80]. The ability to scale up this innovative solution to advance pediatric access of care, improve quality of pediatric care and equity around the globe were evident during the pandemic [81]. Likewise, telemedicine during COVID-19 pandemic provided a good example of how healthcare can adjust to deal with catastrophic events. It can be a tool to increase preparedness and capacity to deal with any disaster.

### Who should Lead in Implementing and Sustaining this Innovative Solution for Many Pediatric Issues?

Governments of many low-income countries might find telemedicine as an appealing model, but bureaucracy and poor infrastructure might be huge barriers [82]. Instantaneously, the success of telemedicine and high demands were encouraging for many global and public health entrepreneurs [83]. The growing global market size of telemedicine is expected to maintain projected annual growth between 13 and 27% worldwide [84]. With lucrative contract to implement telemedicine, entrepreneurs and startups might have different goals and intentions [85]. COVID-19 has paved the way for friendlier regulatory environment for telemedicine around the globe [86]. To hold all entrepreneurs and startups to high ethical standards, telemedicine policies and regulating bodies need to be established in LMIC [87]. The WHO issued guidelines for telemedicine, which can be adopted by many LMICs [88]. Nevertheless, entrepreneurs and startups are needed to advance innovations and overcome many challenges of pediatric telemedicine in many LMIC now and in the future.

### Can Pediatric Telemedicine Cause Harm and be Unethical?

Despite all potentials of telemedicine to improve pediatric care in rural and urban areas of LMIC, it does not come without potential harm. Miscommunication may occur and lead to incorrect diagnoses [89]. The risk of miscommunication might double with international providers offering tele-consults without fully understanding cultural sensitivity and existing resources [90]. When malpractice happens as a product of telemedicine, liability becomes a huge dilemma [91]. Additionally, lack of informed consent of obtaining teleconsultation or using telemedicine might impinge on patients’ autonomy and cause harm [92]. Another possible harm of telemedicine is anticipated to negatively affect continuity of care and provider-patient relationship [93]. Data privacy and breach of confidentially are other major concerns of harm especially for sensitive ages like adolescents [94]. It is worth noting that telemedicine might not be suitable for all pediatric care. Breaking bad news and end-of-life-care requires more human and in-person communication [95]. Overall, telemedicine has many advantages and a few disadvantages to support pediatric care in LMIC that need to be calculated for every unique child and every unique community.

## Conclusion

Telemedicine has the potential to advance pediatric care in rural and remote areas of many LMIC. It offers an additional avenue for providing low-cost universal health coverage for all children. Telemedicine can play a role in lowering under-five mortality by supporting neonatal care, infectious diseases, and NCDs therapies. The quality of pediatric care can be augmented through telemedicine with radiology support, antibiotic stewardship, capacity building and task shifting. Furthermore, it can be scaled up to meet the needs of the children and youths of today and tomorrow. Despite enormous challenges of implementing and sustaining telemedicine in LMIC, existing solutions including low-width band internet, smartphones, instant messaging applications and solar energy can provide new opportunities.

Furthermore, COVID-19 pandemic amplified feasibility, acceptability and accessibility of telemedicine opening a new global market. This global market will attract many entrepreneurs and startups who can advance innovations and increase affordability of telemedicine for many LMIC. At the same time, governments of LMIC need to establish policies and regulating bodies to ensure high quality services and fair access. Telemedicine is here to stay and grow: LMIC should take advantage of this innovation to improve pediatric care and promote equity within their countries.

## Data Availability

All data used to prepare this manuscript were cited and included in this article.

## Abbreviations

LMIC: Low and Middle-Income Countries
NCD: Non Communicable Diseases
ICT: Information and communication technologies
WHO: World Health Organization
mHealth: mobile Health
IM: Instant Messaging
NCD: Non Communicable Diseases
